# Altered glucose-dependent secretion of glucagon and ACTH is associated with insulin resistance, assessed by population analysis

**DOI:** 10.1530/EC-22-0506

**Published:** 2023-03-21

**Authors:** Jan W Eriksson, Reem A Emad, Martin H Lundqvist, Niclas Abrahamsson, Maria C Kjellsson

**Affiliations:** 1 Department of Medical Sciences, Uppsala University, Uppsala, Sweden; 2 Department of Pharmacy, Uppsala University, Uppsala, Sweden

**Keywords:** metabolism, obesity

## Abstract

This study aimed to characterize how the dysregulation of counter-regulatory hormones can contribute to insulin resistance and potentially to diabetes. Therefore, we investigated the association between insulin sensitivity and the glucose- and insulin-dependent secretion of glucagon, adrenocorticotropic hormone (ACTH), and cortisol in non-diabetic individuals using a population model analysis. Data, from hyperinsulinemic–hypoglycemic clamps, were pooled for analysis, including 52 individuals with a wide range of insulin resistance (reflected by glucose infusion rate 20–60 min; GIR_20–60min_). Glucagon secretion was suppressed by glucose and, to a lesser extent, insulin. The GIR_20–60min_ and BMI were identified as predictors of the insulin effect on glucagon. At normoglycemia (5 mmol/L), a 90% suppression of glucagon was achieved at insulin concentrations of 16.3 and 43.4 µU/mL in individuals belonging to the highest and lowest quantiles of insulin sensitivity, respectively. Insulin resistance of glucagon secretion explained the elevated fasting glucagon for individuals with a low GIR_20–60min_. ACTH secretion was suppressed by glucose and not affected by insulin. The GIR_20–60min_ was superior to other measures as a predictor of glucose-dependent ACTH secretion, with 90% suppression of ACTH secretion by glucose at 3.1 and 3.5 mmol/L for insulin-sensitive and insulin-resistant individuals, respectively. This difference may appear small but shifts the suppression range into normoglycemia for individuals with insulin resistance, thus, leading to earlier and greater ACTH/cortisol response when the glucose falls. Based on modeling of pooled glucose-clamp data, insulin resistance was associated with generally elevated glucagon and a potentiated cortisol-axis response to hypoglycemia, and over time both hormonal pathways may therefore contribute to dysglycemia and possibly type 2 diabetes.

## Introduction

Development of type 2 diabetes has been perceived as a process that involves progressive insulin resistance in muscles, adipose tissue, and liver and a gradual failure of pancreatic beta cells to secrete sufficient amounts of insulin to compensate for the resistance. Around 80–90% of patients with type 2 diabetes are overweight (1, 2), and excess adipose tissue, particularly when located viscerally, leads to insulin resistance via mechanisms that are only partially understood (3). The insulin-antagonistic hormone glucagon has been suggested as a key player in the development of type 2 diabetes, along with other hormonal and neural systems (4). Dysregulation of glucagon secretion, in the postprandial state, has been demonstrated in type 2 diabetes and pre-diabetic states (5, 6, 7). Glucagon secretion from pancreatic alpha cells is primarily regulated by the concentration of glucose, being stimulated by hypoglycemia and inhibited by normo- and hyperglycemia. The quantitative importance of other factors, such as inhibition by insulin and stimulation by amino acids, is disputed (8, 9).

Hypoglycemia also triggers the release of other counter-regulatory hormones such as catecholamines, cortisol, and growth hormone (GH) (10). Cortisol secretion is primarily regulated by adrenocorticotropic hormone (ACTH), which, in turn, is stimulated by corticotrophin-releasing hormone (CRH) via the hypothalamic–pituitary–adrenal (HPA) axis. A major physiological effect of these counter-regulatory systems is to raise glucose and alleviate hypoglycemia, but they may also promote insulin resistance and chronic hyperglycemia. Previously, insulin resistance was associated with an altered response of glucagon and the HPA axis to glucose variations (11). Elevated activity in these hormonal pathways may thus contribute to the development of type 2 diabetes. However, the underlying factors are not well understood.

Clamp studies simplify the analyses of complex systems by keeping certain metabolic factors constant while allowing variation in others. Through clamp studies, a general attenuation in the counter-regulatory responses to hypoglycemia after reversal of obesity by a Roux-en-Y Gastric Bypass (RYGB) has been demonstrated (12), and individuals who are overweight and suffer from insulin resistance had an augmented HPA axis response to hypoglycemia (11). Clamp studies are time- and labor-consuming which limits the study size and investigated study conditions, and therefore, they typically have modest statistical power. Mathematical modeling can optimize the utilization of the acquired information through the analysis of pooled data.

Population analysis, as commonly used in pharmacodynamic modeling, is well-suited for pooled data analysis, where data are collected from multiple studies with different study designs. Pharmacodynamic modeling is usually inspired by physiology, as in the turnover (13) and the pool models (14), in which secretion and degradation of a biomarker are driven by stimulation or inhibition by drug concentrations. However, these models can also be used for longitudinal measurements of endogenous hormonal regulation (15, 16, 17, 18, 19, 20) to establish integrative models with high predictability in simulations of not yet performed studies to explain between-subject variability (BSV) by characterization of the impact of covariates, for example BMI and measurement of insulin resistance.

The aim of this work was to characterize the concentration-dependent effects of systemic glucose and insulin on glucagon, ACTH, and cortisol secretion in relation to clinical covariates. This was done through the development of a population model based on data from pooled clamp studies.

## Methods

### Study data and design

Data from three previously published studies were pooled for model development: two hyperinsulinemic normoglycemic–hypoglycemic clamp (henceforth, denoted hypoglycemic clamp) studies in individuals pre- and post-RYGB (12, 21) and a cross-over study with hypo- and hyperglycemic clamps (11). The studies were conducted at Uppsala University (UU) and Uppsala University Hospital (UUH), and study procedures were performed in accordance with the Declaration of Helsinki. All studies were approved by the local Research Ethics Committee of Uppsala (Dnr 2017/550, 2013/480 and 2017/210). Informed consent was obtained from all participants.

All investigations were performed in the morning after an overnight fast. Medical history and anthropometrics were obtained, and baseline blood samples were taken at 8:30 h, directly prior to the clamp experiment. Body fat percentage (BFP) and lean body mass (LBM) were assessed through bioimpedance measurements (Tanita Body Composition, BC-418; Tanita Corporation, Tokyo, Japan). A brief summary of the study designs and participants is included here, whilst detailed descriptions can be found in the original publications (11, 12, 21). Table 1 lists the overall characteristics of the included individuals.

**Table 1:** Baseline characteristics of study participants. Values are presented as medians (25th and 75th percentiles) for all data and by weight class, based on BMI. Blood chemistry is fasting.

	All data	Lean (BMI < 25)	Overweight (25 ≤ BMI < 30)	Obese (BMI ≥30)
*n*	52	11	7	34^a^
*n* per study; Abrahamsson (11)/Almby (20)/Lundqvist (10)	12/11/29^b^	0/0/11	0/0/7	12/11/11^b^
Glucagon, pmol/L	9.79 (7.41–12.4)	8.24 (6.68–10.2)	9.61 (7.64–12.5)	10.5 (7.84–13.5)
ACTH, pmol/L	2.3 (1.35–3.23)^c^	2.3 (2.15–3.35)	2.20 (1.80–2.35)	2.85 (2.30–4.90)^c^
Cortisol, nmol/L	222 (162–271)^d^	255 (249–284)	179 (136–281)	205 (159–257)^d^
Glucose, mmol/L	5.3 (4.8–5.6)	4.8 (4.7–5.2)	5.3 (4.5–5.6)	5.4 (5.0–5.8)
Insulin, mU/L	10.4 (5.50–22.0)	3.80 (2.30–5.25)	8.30 (6.05–9.20)	17.5 (10.5–27.8)
Sex, *n* male/*n* female	7/45	3/8	1/6	3/31
Age, years	40 (30–50)	41 (34–50)	37 (28–49)	41 (30–48)
HbA1c, mmol/mol	34 (32–34.8)	34 (31.5–34)	34 (31.5–36.5)	35.5 (34–36.8)
BMI, kg/m^2^	37.3 (26.5–41.3)	23.0 (22.0–23.5)	27.0 (26.4–28.2)	39.0 (37.5–43.7)
Waist/hip ratio	0.90 (0.84–0.96)^c^	0.83 (0.78–0.87)	0.90 (0.86–0.99)	0.93 (0.89–0.98)^c^
Body fat, *%*	37.0 (27.2–46.7)	22.1 (20.8–28.2)	31.0 (22.0–39.4)	45.3 (37.3–49.0)
GIR_20–60min_, mg/kg-LBM/min	8.13 (4.46–10.8)^e^	13.1 (10.2–15.8)	7.98 (5.72–8.72)	4.85 (3.60–9.84)^e^
HOMA-IR	2.68 (1.40–5.14)	0.910 (0.705–1.24)	1.96 (1.50–2.45)	4.39 (2.64–6.65)

^a^Five individuals had BMI ≥ 30 kg/m^2^ and BMI < 35 kg/m^2^, all from the study by Lundqvist and colleagues (11); ^b^One woman participated only in the hypoglycemic clamp; ^c^ACTH and waist/hip ratios missing for all 12 individuals in Abrahamsson and colleagues (12);^d^One cortisol measurement missing for one male study participant in Almby and colleagues (21); ^e^Bioimpedance missing for one male study participant in Abrahamsson and colleagues (12) GIR_20–60min_ was calculated using an imputed LBM, based on the individual’s weight and the mean body fat % of the male study population.

#### Studies included in pooled analyses

Abrahamsson and colleagues(12) examined symptoms and counter-regulatory responses during hypoglycemic clamps in adults (23–55 years) with a BMI = 36.2–45.2 kg/m^2^, without diabetes before and after an RYGB. Only data from pre-surgery were used for model development.

The Almby and colleagues study (21) was a multi-modal study and explored neurohormonal and cerebral responses, along with cognitive function during hypoglycemia in non-diabetic adults (25–48 years) before and after an RYGB, with BMI = 35.2–45.4 kg/m^2^. The pre-surgery data were used.

The Lundqvist and colleagues study (11) investigated the differences in the secretion of glucose-regulating hormones (i.e. glucagon, ACTH, and cortisol) across a range of glucose concentrations and the impact of being overweight and insulin resistance on the neuroendocrine glucose-regulatory responses. Adults (22–58 years) with a BMI = 19.8–49.5 kg/m^2^, without diabetes were recruited for a cross-over study with hypo- and hyperglycemic clamps.

#### Clamp procedures

**Figure 1:**
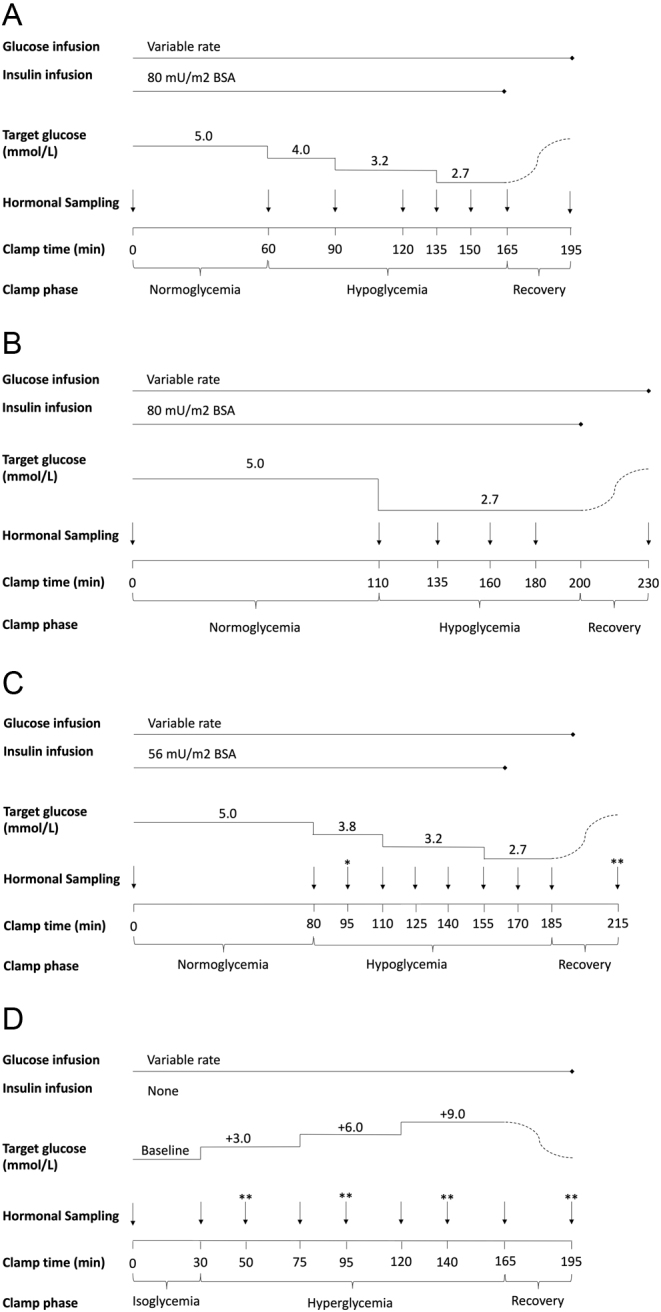
Schematic picture of study designs of hypo- (A–C) and hyperglycemic clamps (D) in Abrahamsson and colleagues (12) (A), Almby and colleagues (21) (B), and Lundqvist and colleagues (11) (C, D). Black vertical arrows indicate the time of measurement of insulin, glucagon, ACTH (not Abrahamsson), and cortisol. Target glucose concentrations are shown at the top of each panel per study/arm. BSA, body surface area. *Neither cortisol nor ACTH sampled at this timepoint, **ACTH not sampled at this timepoint

The designs of the clamps are illustrated in Fig. 1. Briefly, the hypoglycemic clamps were initiated with simultaneous infusions of insulin, potassium chloride (8 mmol/h), and glucose solution (200 mg/mL variable rate). At the start of the experiment, normoglycemia was maintained, targeting 5.0 mmol/L, for 60–110 min after which glucose was lowered to 2.7 mmol/L (stepwise or in one step). Hypoglycemia was maintained for up to 105 min and was followed by a 30-min recovery when insulin infusions were terminated while glucose was infused at a fixed rate (100–200 mg/kg/min). The average glucose infusion rate (in mg/min) per kg-LBM from 20–60 min of normoglycemia (GIR_20–60min_) was used to reflect insulin sensitivity, that is the inverse of insulin resistance.

The hyperglycemic clamp commenced with a 30-min isoglycemic phase, during which glucose was kept at the fasting concentration, with or without a variable rate glucose infusion (200 mg/mL). Glucose was increased stepwise in 45-min periods by 3, 6, and 9 mmol/L above isoglycemia. Thereafter, the infusion was terminated to allow glucose to return to normal range.

#### Bioanalytical methods

In all experiments, glucose was sampled every 5 min from an arterialized vein and analyzed with a Contour glucometer (Bayer Healthcare, Leverkusen, Germany). Fasting glucose (hexokinase method) and HbA1c were analyzed at the Department of Clinical Chemistry at UUH. Hormones were sampled at the start and end of the normoglycemic phase and at regular intervals (15–30 min) throughout the hypoglycemic/hyperglycemic phases as depicted in Fig. 1. Hormonal samples were analyzed immediately or frozen at −80°C for later analysis. Insulin, cortisol, and ACTH (except Abrahamsson (11)) were analyzed at the Department of Clinical Chemistry at UUH using immunoassays (CobasE, Roche for insulin and cortisol; Immulite 2000XPi, Siemens Healthcare Global for ACTH). Glucagon was analyzed using an ELISA (#10-1271-01, RRID:AB_2737304, Mercodia, Uppsala, Sweden; within-assay CV 2.1–14%, between-assay CV 7.0–16%) at the Clinical Diabetes and Metabolism Research Laboratory, UU.

### Population modeling

Turnover and pool models were developed for the counter-regulatory hormones, that is glucagon, ACTH, and cortisol, across all individuals and studies. The turnover models (Eq. 1) were parameterized with *C* – the plasma concentration of glucagon, ACTH, or cortisol, *C*_0_ – the initial plasma concentration of glucagon (*Gn*_0_), ACTH (*ACTH*_0_), or cortisol (*Cort*_0_), *k_secr_* – the zero-order secretion rate of the hormone, and *k_degr_* – the first-order degradation rate constant of the hormone (13).


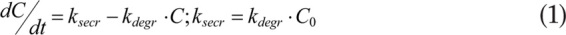



Fasting glucose and insulin were required for the initial conditions of the model equations and data of individuals missing this information were excluded. The pool models (Eqs. 2 and 3) were parameterized with *C_ex_* – the extravascular concentrations of glucagon, ACTH, or cortisol, *k_synth_* – the zero-order synthesis rate of the extravascular hormone, *k_secr_* – the first-order secretion rate constant from extravascular to plasma and the above-defined parameters of the turnover model, *C*, *C*_0_, and *k_degr_* (14).


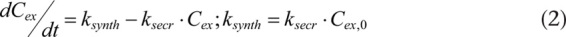




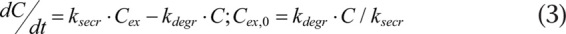



Dynamics in the models were driven by stimulants, in this case plasma glucose, plasma insulin, and/or ACTH, stimulating, through an *E_max_* function or inhibiting, through an *I_max_* function, the synthesis, secretion or degradation of the hormones. A sigmoidicity term, that is the Hill factor (γ) was explored in the *E_max_* and *I_max_* functions. To allow for additional delay between stimulant and hormone, the stimulant concentrations were allow additional delayed an effect compartment model (22) (Eq. 4) was also explored, where *t*_1/2_ is the half-life of delay of stimulant, *S* is the plasma concentration of stimulant, and *S_e_* is the delayed concentrations of the same.


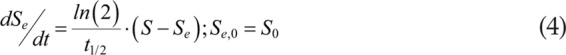



#### Glucagon sub-model

Glucagon dynamics were primarily modeled as driven by plasma glucose, and the subsequent contribution of plasma insulin was explored. Initially, a turnover model was used to investigate the relationship between glucose and insulin effects, implemented as below (Eqs. 5–8).


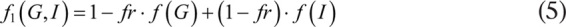




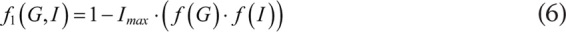




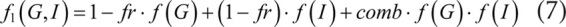




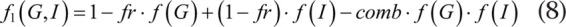



Thereafter, the turnover model was compared to a pool model, investigating the effect of glucose and/or insulin on *k_secr_* or *k_synth_*. In the last step, the effects of delaying the action of glucose and/or insulin were explored with an effect compartment model.

#### ACTH sub-model

ACTH dynamics were primarily modeled as driven by plasma glucose with a sigmoidal *I_max_* function, comparing the turnover and pool models with dynamics on *k_secr_* or *k_synth_*. The dynamics were investigated as driven by plasma glucose as well as delayed, effect compartment glucose. The last modeling step was to explore negative feedback of cortisol on ACTH after the cortisol sub-model had been developed.

#### Cortisol sub-model

Cortisol dynamics were modeled as primarily driven by ACTH with a sigmoidal *E_max_* function, comparing the turnover and the pool models, with dynamics on *k_secr_* or *k_synth_*. This was explored with both direct and delayed ACTH. The last modeling step was to explore the direct effects of glucose on cortisol.

#### Covariates

After finalizing all three sub-models, covariates were investigated as factors explaining BSV. Missing data of baseline covariates were imputed as the median of the population. The investigated covariates were age, GIR_20–60min_, sex, HOMA-IR, BMI, waist/hip ratio, BFP, fasting plasma glucose, and HbA1c and were tested through either linear or exponential additions to parameters with BSV. Due to the small study size, a robust covariate selection was performed with four-fold cross-validation (XV) step-wise covariate modeling (SCM) (23) and full random effect modeling (FREM) (24).

#### Statistical assessments during model-building and model-fit evaluation

Measurements below limit of quantification (BLQ) were handled with an additional residual error, thereby allowing more uncertainty in these observations. Additions of structural elements, parameters, and relationships with covariates were only included if a better fit to the data was indicated based on the difference in objective function value between two competing models (ΔOFV). The likelihood ratio test was used for nested models with β = 0.05 and the Akaike information criterion was used for non-nested models. The standard errors (SE) of the parameter parameters and BSV were obtained through the use of bootstrap simulations (*n* = 200). A good predictive performance of the model was assessed by the use of visual predictive checks.

Random variables for BSV were initially implemented on parameters of baseline measurements and thereafter added to the model if indicated by the above-described model selection criteria. Random variables of between-occasion variability (BOV) were investigated only on parameters of baseline measurements.

Data management, statistical calculations, and graphical evaluation were performed in R v.4.0.3 (R Foundation for Statistical Computing, Vienna, Austria) (https://www.R-project.org/). Simulations and estimations were performed using NONMEM v.7.4.4 (Icon Development Solutions, Ellicott City, MD, USA) (25) and XV-SCM and FREM were used as implemented in PsN v.5.2.0 (26).

## Results

### Summary of data

Of the original 53 individuals, 52 were included in the pooled data. One individual was excluded as the fasting insulin was missing. Table 1 summarizes covariates and baseline measurements of glucose and hormones. Investigated glucose and insulin concentrations ranged 1.9–16 mmol/L and 0.8–580 µU/mL, respectively, while glucagon, ACTH, and cortisol ranged 0.2–96 pmol/L, 1.0–73 pmol/L, and 73–819 nmol/L, respectively (Table 1).

### Population data analysis

#### Glucagon sub-model

**Figure 2:**
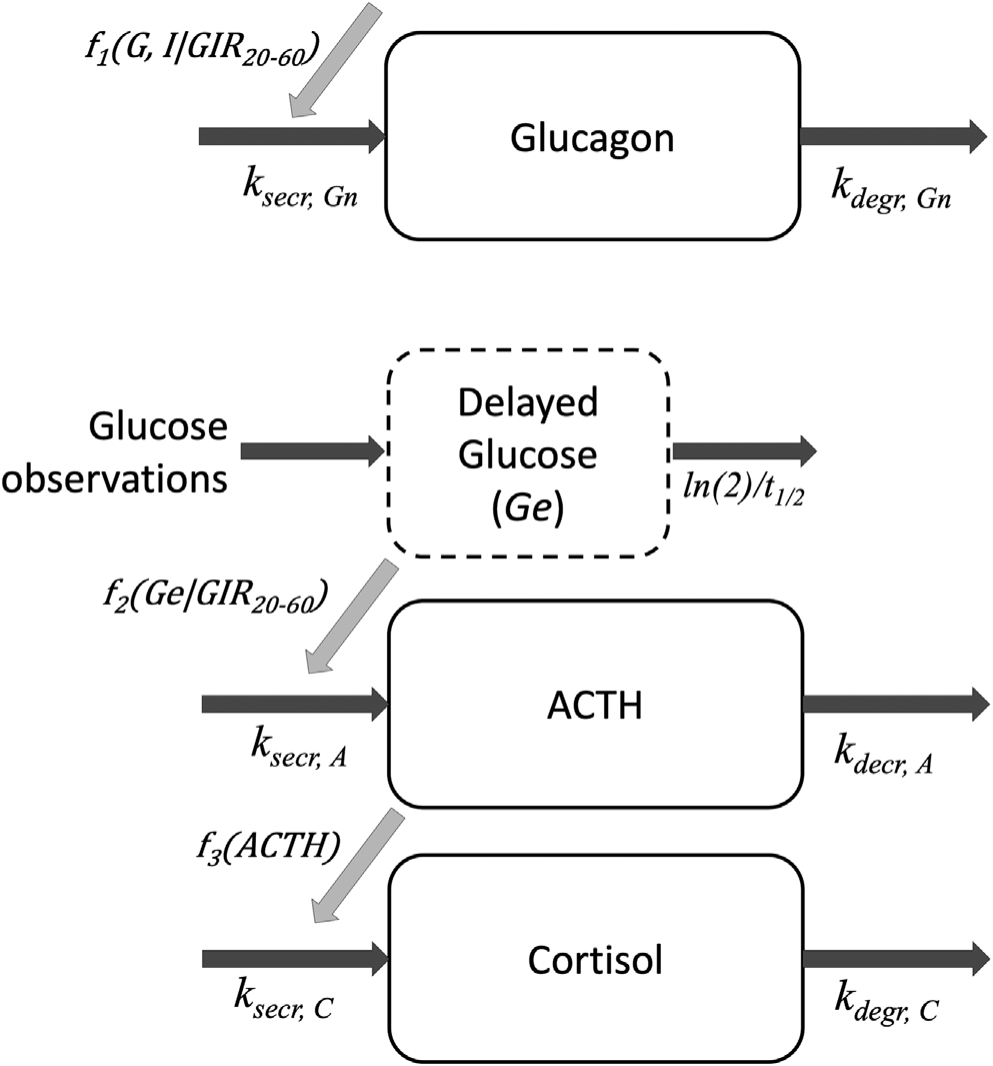
Schematic picture of the final sub-models of glucagon, ACTH, and cortisol. Dark gray arrows indicate mass transfer while light gray arrows indicate the location of the stimulus for dynamics. *k_secr_*, secretion rate; *k_degr_* degradation rate constant; *t_1/2_*, half-life of glucose delay, *f_1_(G,I|GIR_20–60min_)*, the function of glucose and insulin affecting glucagon, where insulin effect is influenced by the glucose infusion rate at normoglycemia (GIR_20–60min_);* f_2_(*Ge*|GIR_20–60min_),* the function of delayed glucose affecting ACTH, where the effect is influenced by GIR_20–60min_; *f_3_(ACTH),* the function of ACTH affecting cortisol.

**Figure 3:**
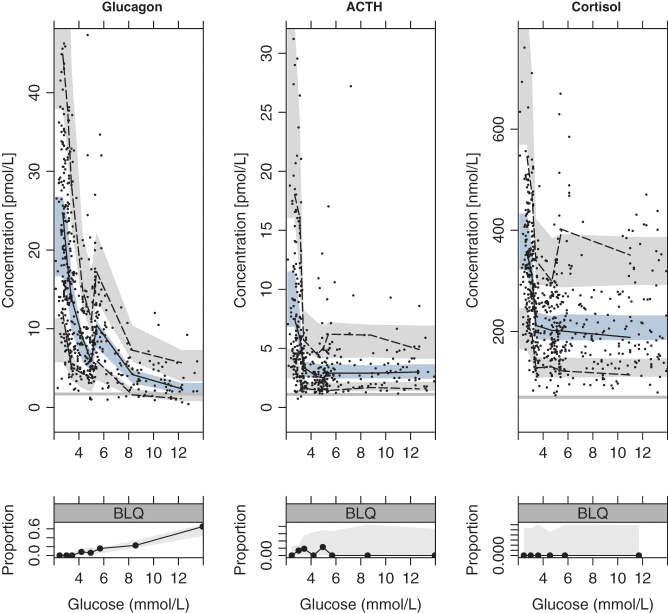
Visual predictive check of glucagon (left), ACTH (middle), and cortisol (right) vs glucose concentrations. The top panels show the concentrations of variables, while the bottom panels show the proportion of measurements below limit of quantification (BLQ). The median (solid line) and 2.5th and 97.5th percentiles (dashed lines) of the observations (indicated with dots) are overlayed with the 95% confidence interval of the corresponding percentiles of the simulated data (shaded area, 1000 simulations). The gray horizontal line indicates the limit of quantification.

**Figure 4:**
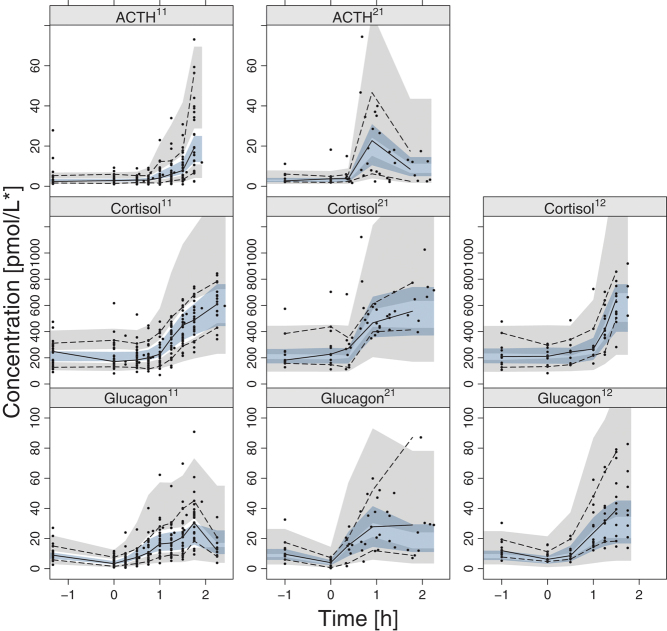
Visual predictive check of ACTH (top), cortisol (middle), and glucagon (bottom) in the studies by Lundqvist (11) (left), Almby (21) (middle), and Abrahamsson (12) (right) vs time, with time = 0 being the start of the hypoglycemia in each study. The median (solid line) and 2.5th and 97.5th percentiles (dashed lines) of the observations (indicated with dots) are overlayed with the 95% confidence interval of the corresponding percentiles of the simulated data (shaded area, 1000 simulations).

**Figure 5:**
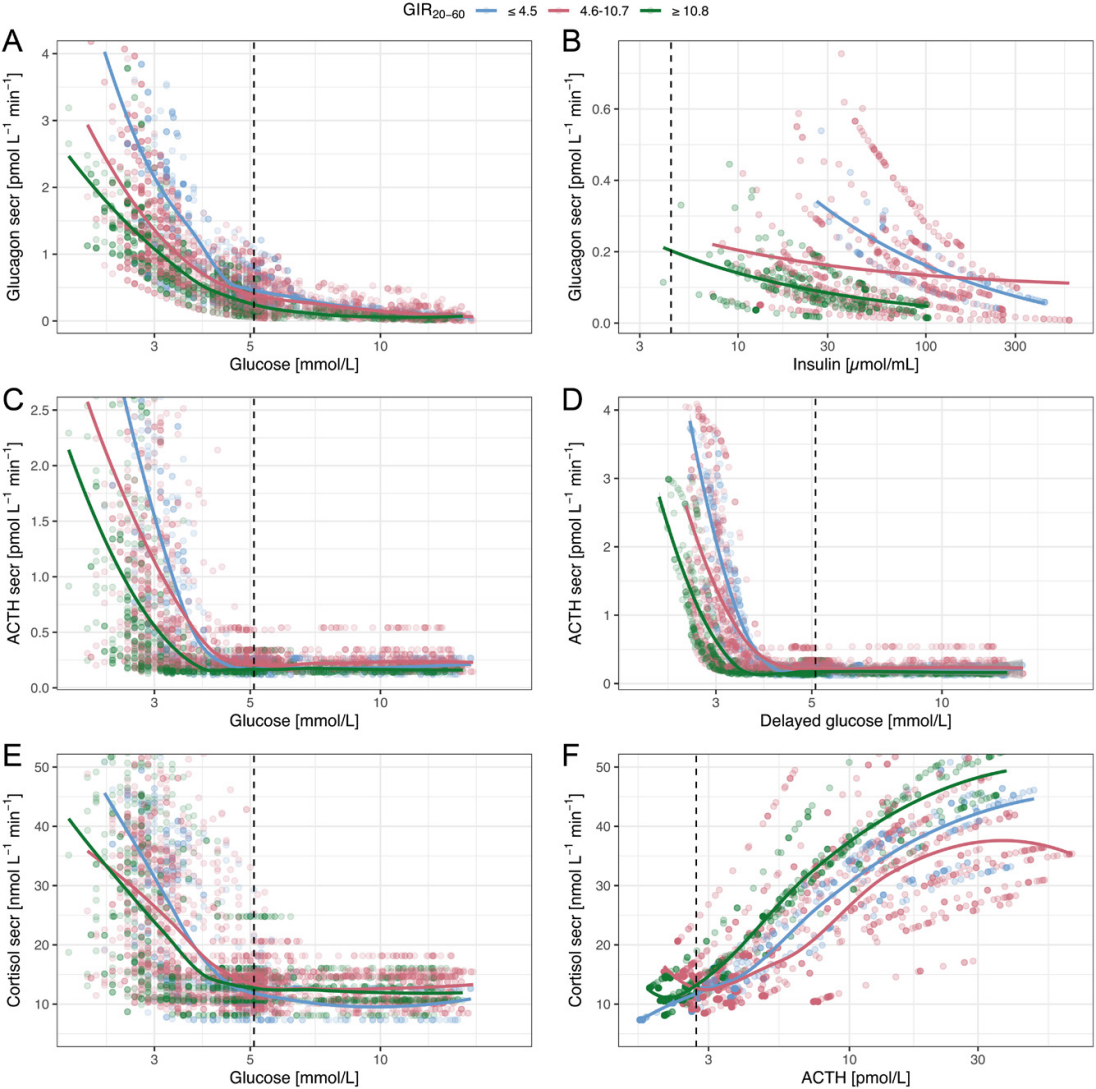
Secretion of glucagon (A), ACTH (B), and cortisol (C) vs glucose as well as glucagon secretion vs insulin (D), ACTH vs delayed glucose (E), and cortisol vs ACTH (F). Points represent individual model predictions, colored by GIR_20–60min_ and the solid colored, lines are smooths through the data, grouped by GIR_20–60min_. The black dashed, vertical line indicates the median of the x-axis variable of individuals with BMI ≤ 25 kg/m^2^ at baseline, that is 5.1 mmol/L glucose, 4.4 µU/mL insulin, or 2.7 pmol/L ACTH.

Independent of the relationship to plasma glucose, the inclusion of plasma insulin resulted in major improvements in model fit. The multiplicative inhibition of glucose and insulin on glucagon (Eq. 6) had the smallest improvement in OFV (∆OFV = −312). The remaining three relationships (Eq. 5, 7 and 8) had similar fit (∆OFV = −355), favoring the additivie inhibiton relationship (Eq. 5), as it had one parameter less than the other relationships. Glucose and insulin, acting on the synthesis of glucagon, in a pool model resulted in a similar fit to the turnover model (∆OFV = −0.8). However, moving the effect of glucose and insulin to secretion in the pool model resulted in a worse fit (∆OFV = 108). Removing the Hill factor of the *I_max_* function of glucose significantly deteriorated the fit of the model to the data (∆OFV = 947), and the Hill factor was retained in the model. Removing the Hill factor of the insulin relationship did, however, not worsen the fit. Allowing additional delay of either glucose or insulin had no effect on the model fit. The final glucagon sub-model is shown in Fig. 2, and the performance of the final model vs glucose and time is shown in Fig. 3 and 4, respectively. The effect of glucose and insulin is described by equation 9. BSV was implemented on baseline glucagon and both potency parameters (*IC_50,G-G_* and *IC_50,G-I_*). BOV significantly improved the fit when added to the baseline.


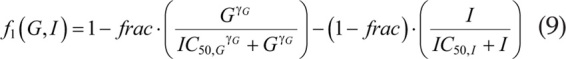



where the glucose effect is described by *G* – glucose concentrations, *frac* – the fractional effect attributed to glucose in absence of insulin, *γG* – the Hill factor, and *IC_50, G_* – the potency of glucose on glucagon. The insulin effect is described by *I* – insulin concentrations, *IC_50, I_* – the potency of insulin on glucagon, and *1-frac* – the fractional effect attributed to insulin in absence of glucose. The fit of the model to the data was excellent with a good predictive performance of both the glucagon data and proportion of data BLQ (Fig. 3– left panels) with 18.9% of the residuals outside the 80% confidence interval (CI), evenly distributed around the median. Figure 5A illustrates the inhibition of glucagon secretion by glucose and insulin. At the start of the study, in fasting conditions, the suppression of glucagon varied greatly in the population (90% CI: 68–95%) related to the high BSV in *IC_50, I_* (Table 2). This high BSV and the variable fasting insulin also explain the high variability of fasting glucagon.

**Table 2:** Table of parameter estimates of the final model. Presented with typical parameter of population, between-subject (BSV) and between-occasion variability (BOV); the last two reported as coefficient of variation in %. All parameters are reported with relative standard error (RSE) in %.

Parameter	Description	Typical parameter (RSE)	BSV (RSE)	BOV (RSE)
*Gn_0_*	Baseline glucagon concentration (pmol/L)	8.35 (2.38)	39.6 (5.60)	20.0 (5.42)
*k_out, G_*	Removal rate of glucagon (min^−1^)	0.0612 (6.29)	-	-
*Frac*	Fraction of glucose contribution to glucagon inhibition	0.750 (2.35)	-	-
*IC_50,G_*	Potency of glucose on glucagon secretion (mmol/L)	2.91 (3.02)	23.4 (9.22)	-
*γ_G_*	Shape factor of glucose effect on glucagon	5.26 (3.94)	-	-
*IC_50,I,GIR=8.13_*	Potency of insulin on glucagon secretion (µU/mL)	5.51 (11.3)	93.2 (6.50)	-
*GIR_IC50,I_*	Effect of GIR_20–60min_ on insulin potency (% mg/kg/min)	−15.7 (19.3)	-	-
∑*_G_*	Residual error of glucagon (%)	33.8 (5.07)	-	-
*ACTH_0_*	Baseline ACTH concentration (pmol/L)	2.94 (4.76)	29.4 (13.0)	8.10 (11.4)
*k_out, A_*	Removal rate of ACTH (min^-1^)	0.0709 (14.5)	-	-
*t_1/2_*	Half-life of delay of glucose effect on ACTH (min)	8.68 (6.56)	-	-
*I_max,A_*	Maximal glucose effect on ACTH	0.941 (1.11)	-	-
*IC_50,A,GIR=8.13_*	Potency of glucose on ACTH secretion (mmol/L)	2.85 (1.70)	7.76 (6.96)	-
*γ_A_*	Shape factor of glucose effect on ACTH	20.9 (8.74)	-	-
*GIR_IC50,A_*	Effect of GIR_20–60min_ on glucose potency (% mg/kg/min)	−1.55 (16.4)	-	-
∑*_A_*	Residual error of ACTH (%)	38.1 (10.7)	-	-
*Cort_0_*	Baseline cortisol concentration (nmol/L)	207 (3.60)	25.1 (9.55)	-
*k_out, C_*	Removal rate of cortisol (min^-1^)	0.0608 (9.20)	-	-
*Emax_C_*	Maximal ACTH effect on cortisol secretion	41.8 (7.40)	-	-
*IC_50,C_*	Potency of ACTH on cortisol secretion (pmol/L)	6.90 (5.12)	-	-
*γ_C_*	Shape factor of ACTH effect on cortisol	1.29 (2.30)	-	-
∑*_C_*	Residual error of cortisol (%)	29.3 (5.79)	-	-
*f_Add_*	Multiplicative factor of residual error of data BLQ	2.01 (7.75)	-	-

BLQ, below limit of quantification.

#### ACTH sub-model

The turnover and the pool model with effects on *k_synth_* had similar fit (∆OFV = −1.7 for pool model), favoring the turnover model with fewer parameters. The pool model with effect on *k_secr_* performed worse than all other investigated models (∆OFV = 592) and was thus not further explored. Delaying glucose concentrations through an effect compartment model (Eq. 4) improved the fit of the model to the data further (∆OFV = −9.4). Removing the Hill factor of the *I_max_* function deteriorated the fit significantly (∆OFV = 305). The final relationship between ACTH secretion and glucose was best described as in equation 10. The BSV was implemented on baseline ACTH and glucose potency (*IC_50,A_*), and BOV significantly improved the fit when implemented on the baseline. Negative feedback of cortisol on ACTH resulted in a highly unstable model, and further model refinements related to the feedback were abandoned due to parameter unidentifiability.


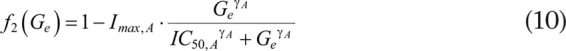



where the effect is described by *G_e_* – the delayed glucose concentrations, delayed by t_1/2_ – the half-life of delay (Eq. 4), *I_max,A_* – the maximal effect of glucose on ACTH, *_γA_* – the Hill factor, and *IC_50,A_* – the potency of glucose on ACTH. The fit of the model to the data was fair with a good predictive performance of both the ACTH data and proportion of data BLQ (Fig. 3– middle panels) with 15% of the residuals outside the 80% CI, evenly distributed around the median. Figure 5B shows the relationship between ACTH secretion and glucose. The maximum inhibition (*I_max,A_*) was close to 1 (0.96, Table 2); however, setting ACTH suppression to 1 deteriorated the model (∆OFV > 17,000). The inhibition of ACTH at fasting was similar between all individuals in the population (90% CI: 95.51–95.53%).

#### Cortisol sub-model

ACTH driving a turnover or a pool model with effects on *k_synth_* performed similarly in describing cortisol (∆OFV = −0.06 for pool model), favoring the turnover model with fewer parameters. The pool model with effect on *k_secr_* performed worse than the other investigated models (∆OFV = 369). Delaying ACTH through an effect compartment model did not improve the fit of the model significantly (∆OFV = −0.4). Although the estimated Hill factor of the *E_max_* function was close to 1, removing the parameters deteriorated the fit significantly (∆OFV = 28). The final model of cortisol is shown in equation 11. The BSV was added to baseline cortisol and the addition of BOV did not improve the fit. Glucose effects, additional to ACTH, on cortisol resulted in a highly unstable model, indicating parameter unidentifiability.


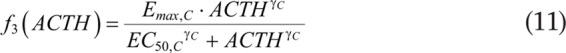



where the effect is described by *ACTH* – the ACTH concentrations, *E_max,C_* – the maximal effect of ACTH on cortisol, *_γC_* – the Hill factor, and *EC_50,C_* – the potency of ACTH on cortisol. The fit of the model to the data was excellent with a good predictive performance of both the cortisol data and proportion of data BLQ (Fig. 3– right panels) with 18.9% of the residuals outside the 80% CI, evenly distributed around the median. The relationship between cortisol secretion and glucose and ACTH is shown in Fig. 5C.

#### Impact of covariates on model parameters

According to the XV-SCM, the significant relationships were GIR_20–60min_ on glucose *IC_50_* of ACTH (identified in two data-splits), with a secondary relationship of GIR_20–60min_ or BMI on insulin *IC_50_* of glucagon (identified in one data-split each). The inability to separate between GIR_20–60min_ and BMI was expected given the high correlation between BMI and GIR_20–60min_ (corr=-56%).

With FREM, the four strongest correlations (≥ 50%) between parameter and covariates were: 1) GIR_20–60min_ on glucose *IC_50_*of ACTH (corr = −56%), 2) BMI on insulin *IC_50_* of glucagon (corr = 53%), 3) HOMA-IR on glucose *IC_50_* of ACTH (corr = 51%), and 4) GIR_20–60min_ on insulin *IC_50_* of glucagon (corr = −50%). Correlations between BMI/GIR_20–60min_ and HOMA-IR/GIR_20–60min_ were high: −56% and −69%, respectively.

An SCM, with a 5% significance level of forward inclusion and a 1% significance level of backward elimination, resulted in the inclusion of two relationships: GIR_20–60min_ on insulin *IC_50_* of glucagon and GIR_20–60min_ on glucose *IC_50_* of ACTH.

All covariate methods (XV-SCM, FREM, and SCM) indicated only relationships with insulin *IC_50_* of glucagon and glucose *IC_50_* of ACTH. GIR_20–60min_ came across as a strong covariate for the relationship with ACTH; all other covariates were ranked secondary. GIR_20–60min_ was also a strong predictor for the relationship with glucagon, although BMI was often ranked similarly. Combining the results of all covariate methods, considering the convenience of model predictions, GIR_20–60min_ was chosen as the sole predictor in the final model. The relationships were as described in equations 12 and 13.


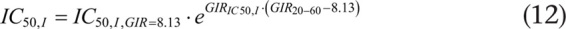




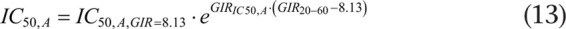



Where IC_50,I,GIR=8.13_ and IC_50,A,GIR=8.13_ are the potencies of insulin on glucagon and glucose on ACTH, respectively, for an individual with GIR_20–60min_=8.13 mg/kg-LBM/min. GIR_IC50,I_ and GIR_IC50,A_ are the covariate effects of GIR_20–60min_ on the IC_50_:s.

**Figure 6:**
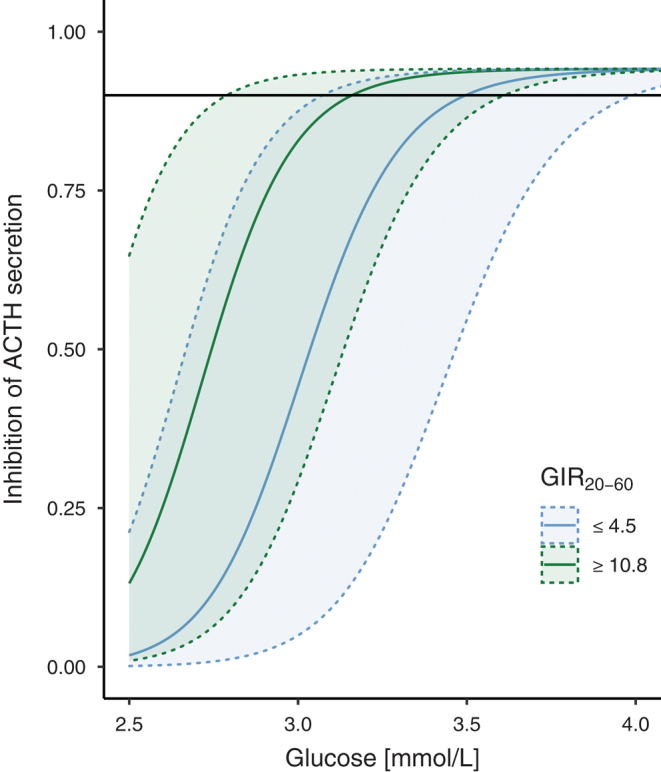
Inhibition of ACTH secretion by glucose, illustrated for insulin-sensitive (GIR_20–60min_ ≥ 10.8 mg/kg-LBM/min; green) and insulin-resistant (GIR_20–60min_ ≤ 4.6 mg/kg-LBM/min; blue) individuals. The blue and green solid lines indicate the medians and the 90% confidence interval is shown as a shaded area. The horizontal line indicates 90% inhibition.

GIR_20–60min_ varied greatly, and the interquartile range of GIR_20–60min_ was 4.5–10.8 mg/kg-LBM/min. The upper and lower quartiles of the pooled cohort were used in figures to illustrate insulin-sensitive (GIR_20–60min_ ≥ 10.8 mg/kg-LBM/min) and insulin-resistant (≤4.5 mg/kg-LBM/min) individuals, respectively. Thus, the study population was divided into three groups for strictly illustrative purposes. As illustrated in Fig. 6, individuals with high insulin sensitivity had sustained ACTH suppression at lower glucose concentrations compared to individuals with insulin resistance. The glucose concentration required for 90% inhibition of ACTH was 3.1 mmol/L (90% prediction interval ranged 2.7–3.6 mmol/L) and 3.5 mmol/L (3.1–4.0 mmol/L) for insulin-sensitive and insulin-resistant individuals, respectively.

**Figure 7:**
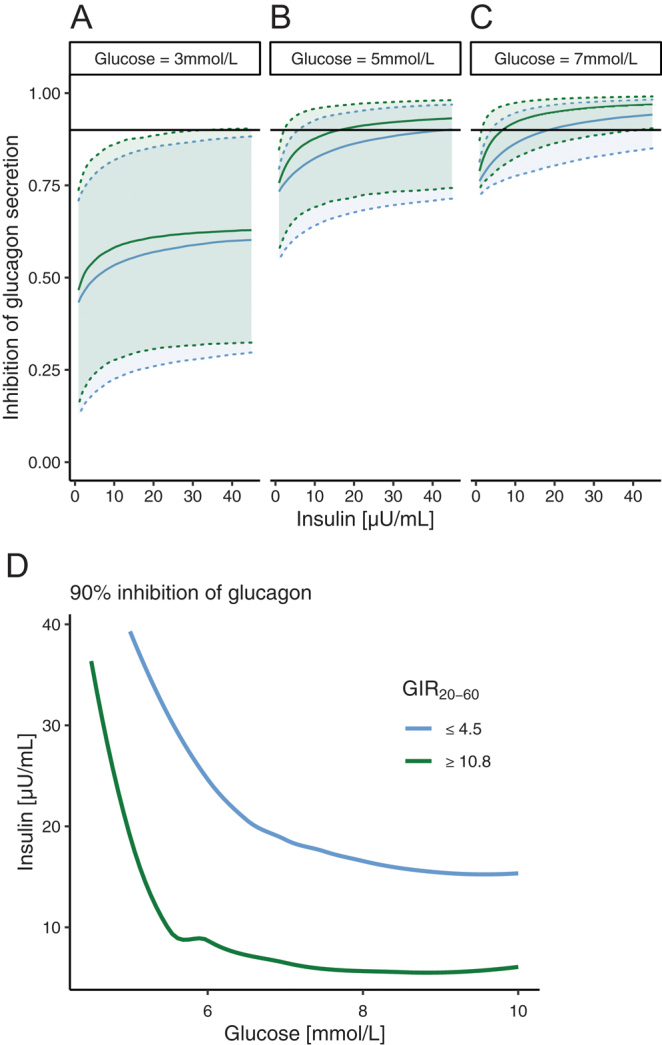
Inhibition of glucagon secretion by insulin for (A) hypo-, (B) normo-, and (C) hyperglycemia as well as (D) the relationship between glucose and insulin for 90% inhibition of glucagon, illustrated for insulin-sensitive (GIR_20–60min_ ≥ 10.8 mg/kg-LBM/min; green) and insulin-resistant (GIR_20–60min_ ≤ 4.6 mg/kg-LBM/min; blue) individuals. The blue and green solid lines indicate the medians, and the 90% confidence interval is shown as a shaded area. The horizontal line indicates 90% inhibition.

For glucagon inhibition, the impact of GIR_20–60min_ was found on the relationship between glucagon and insulin (Fig. 7). In individuals with normal insulin sensitivity, sustained glucagon suppression was seen with lower insulin concentrations compared to individuals with insulin resistance, and glucagon is thus not suppressed to the same extent for high insulin concentrations in individuals with insulin resistance. Consequently, fasting glucagon was elevated for individuals with insulin resistance due to insufficient glucagon suppression from insulin at normoglycemia. The insulin concentration required for 90% inhibition of glucagon at 5 mmol/L glucose was 16.3 and 43.4 µU/mL for insulin-sensitive and insulin-resistant individuals, respectively. The glucose-insulin concentrations required for 90% glucagon inhibition clearly shifted downwards in insulin-resistant individuals (Fig. 7D, green line). The code for the final population model is available on Github (https://github.com/miakjellsson/GN_ACTH_Cor).

## Discussion

In this work, data from three previous clamp studies were pooled (11, 12, 21), exploring a wide glucose range, from profound hypoglycemia to marked hyperglycemia. A population model-based analysis was used to quantify determinants of glucose- and insulin-dependent counter-regulatory hormone secretion. It was hypothesized that individuals who are prone to type 2 diabetes, as reflected by insulin resistance, have an elevated glycemic setpoint for homeostatic regulation and that this may be important for the progression to type 2 diabetes.

As a data-driven modeling approach was used, the final model of glucagon, ACTH, and cortisol represented the best fit for the pooled data. The benefit of a data-driven approach is that model attributes are included only when supported by data, rendering a robust and parsimonious model. The disadvantage is that the resulting model structure is dependent on the information in the data. Consequently, the model structure of a biological system, that is the HPA axis, may differ between different publications. Several population models of glucagon, ACTH, and cortisol have previously been published (16, 17, 20). The model diversity illustrated this phenomenon. It is, however, reassuring that the main model structure of the final model was similar to other published models (16), with the main differences being the lack of circadian rhythm (16) and the insulin resistance effects.

As all data used in this study were collected in the morning, without longer duration of collection, estimation of circadian rhythm was not feasible, which limits the use of the model for whole-day simulations. Combination of the circadian rhythm from other models, for example the ACTH model by Lönnebo and colleagues (16) combined with the final model presented, could resolve this limitation.

The main strength of the pooled data was the range of between-subject insulin resistance and the within-subject glucose and insulin concentrations, which has not been previously modeled. However, as the population size was limited, and to reduce the risk of over-interpretation, XV-SCM and FREM (23, 24) were utilized to identify parameters associated with insulin resistance and/or obesity.

**Figure 8:**
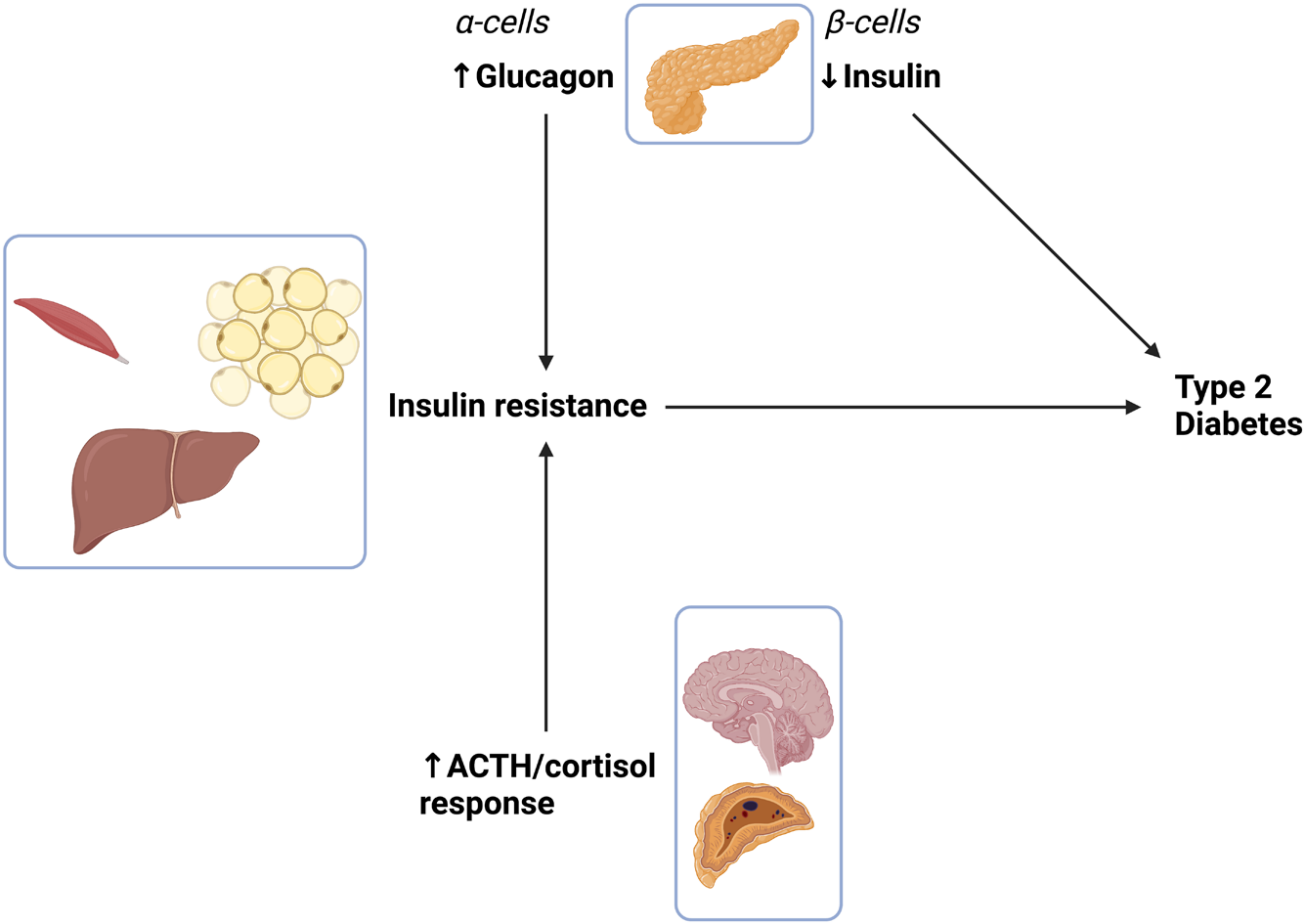
Hypothesized contribution of general hyperglucagonemia and augmented ACTH/cortisol responses to the development of insulin resistance and type 2 diabetes. Created with Biorender.com.

For glucagon, insulin resistance was associated with a shift in estimated *IC_50_* of insulin and was similar to the fasting insulin for insulin-sensitive individuals with a GIR_20–60min_ ≥ 10.8 mg/kg-LBM/min of 5.1 µU/mL. Thus, insulin resistance impacted fasting glucagon through insulin. However, whether GIR_20–60min_ or BMI is the best predictor requires further investigation. For ACTH, insulin resistance was associated with a shift in *IC_50_* of glucose, while cortisol dynamics were accounted for by ACTH. Thus, a change in the hypoglycemic threshold for activation of counter-regulatory responses in individuals with insulin resistance was supported. The estimate of *IC_50_* of glucose (2.85 mmol/L) on ACTH was similar to the cut-off for pathological hypoglycemia of 3 mmol/L (27). Exaggerated ACTH/cortisol responses can, therefore, potentially contribute to aggravated insulin resistance and long-term glucose elevation. Taken together, the increased glucagon secretion across a wide glycemic range and the increased cortisol axis activity in the hypoglycemic and normoglycemic range are both likely contributors to insulin resistance and potentially to type 2 diabetes development, as illustrated in Fig. 8.

### Glucagon

Lundqvist and colleagues (11) found no difference in hypoglycemic glucagon secretion *per se* between overweight and lean individuals, although less hyperglycemia-induced suppression was observed in overweight individuals. The present pooled analysis corroborates previously reported associations between insulin resistance and hyperglucagonemia (28, 29) and extends the validity of these observations to a broad glycemic range 28, 29 (1.9–16 mmol/L). Moreover, the analysis suggests that hyperglucagonemia was driven by individual differences in insulin-mediated suppression of glucagon independent of glucose levels. This may be related to insulin resistance of pancreatic alpha cells which has been demonstrated in type 2 diabetes (30). Information about intra-islet insulin concentrations would be of interest. However, obtaining such measures *in vivo* is challenging and it was not available in this study. Morettini and colleagues (31) developed a model of glucagon, predicting local insulin concentrations from C-peptide measurement in an oral glucose tolerance test. Given the different study conditions, parameters are not directly comparable between the models. Apart from study conditions, Moretti’s model includes an insulin stimulation of elimination of glucagon, while our model contains an insulin inhibition of production of glucagon. However, these approaches are essentially the same mathematically.

### Cortisol axis

A major finding from this pooled analysis was that the insulin-resistant population had a shifted response of the cortisol axis to hypoglycemia; 50% response in ACTH was achieved at 3.0 mmol/L for the insulin-resistant population while the same was achieved at 2.7 mmol/L for the insulin-sensitive population. Glucose-stimulated ACTH secretion may be directly mediated through ACTH-producing pituitary cells or indirectly via hypothalamic glucose-sensing neurons that modulate the secretion of CRH, which in turn stimulates pituitary ACTH secretion (32, 33). The latter was supported by the final models estimated delay of glucose-stimulus on ACTH. It is, therefore, likely that glucose-sensing and regulation by the hypothalamus, and potentially other brain regions, contribute to the elevated responses of the cortisol axis during hypoglycemia among individuals with insulin resistance. When assessing the plasma glucose–ACTH relationship, the combination of the delayed glucose and the steepness of the relationship (as indicated by the Hill factor) creates a model behavior, where hypoglycemic response of ACTH (plasma) is delayed, creating an apparent lag-phase between the glucose lowering and the stimulation of ACTH secretion.. Although some caution is warranted regarding the transferability of these results to the real-world setting, the fact that the glycemic threshold for ACTH response was within the physiologically plausible range for insulin-resistant individuals does imply importance for the day-to-day glucose metabolism in these individuals. The cortisol axis response was associated with the GIR_20–60min_ through ACTH and was independent of BMI in the final model, which points to a potential role of ACTH in the development of insulin resistance. However, longitudinal studies of larger cohorts, designed for this purpose, would be required to support this hypothesis.

The dynamics of cortisol secretion were found to be fully explained by ACTH-stimulus, suggesting an absence of ACTH-independent pathways for the regulation of cortisol secretion under the employed experimental conditions. Such pathways have been proposed, including stimulation by catecholamines (34) and cytokines (35, 36). While these pathways may explain the dissociation of cortisol and ACTH relationships in critical illness and post-surgery (35, 36), they have most likely limited importance in this context.

### Other neuroendocrine factors

Additional glucose-elevating neuroendocrine pathways, such as the GH axis and the sympathoadrenergic system, may be important in the development of insulin resistance. Insulin resistance has been found previously to be associated with a less dynamic hypoglycemic autonomic nervous system response, characterized by both a smaller inhibition of parasympathetic nerve activity (PASY) and smaller activation of sympathetic nerve activity (SY) (11). Visceral adiposity and insulin resistance have been linked to an increased SY/PASY activity ratio during normoglycemic conditions (37, 38, 39). The attenuated dynamics of SY and PASY activity during hypoglycemia in individuals who are overweight and insulin-resistant may potentially contribute to elevated glucose, although mechanisms are not understood.

We and others have previously shown that GH is reduced in obesity but rises upon weight loss (11, 40). Likewise, the hypoglycemic GH response increases following gastric bypass surgery (12, 21), which paradoxically would be expected to promote elevation of glucose concentration.

Taken together, many neuroendocrine pathways may contribute to altered responses to glucose fluctuations in individuals with insulin resistance, but their respective roles need further validation in large prospective cohorts. It was previously reported that elevated counter-regulatory neuroendocrine responses to hypoglycemia operate in type 2 diabetes (41), and this may support their contribution to the development and/or maintenance of long-term hyperglycemia. Of note, the current work does not include modeling of insulin secretion, as this is well-studied (42).

An overview of proposed crosstalk between endocrine organs is shown in Fig. 8. With regards to inference about causality, we acknowledge that the current analysis is cross-sectional and does not prove that the demonstrated hormonal dysregulations lead to insulin resistance, albeit this appears likely. Hormonal perturbations may instead be consequences of insulin resistance or its associated conditions. Longitudinal cohort studies, ideally including specific interventions, would shed light on causality.

### Limitations

Albeit data were pooled from three clamp studies, the sample size in this work is limited. The data are cross-sectional, and larger, longitudinal and interventional studies are needed to establish a contribution by glucagon and HPA axis dysregulation in the development of insulin resistance and type 2 diabetes. Furthermore, we only have data of plasma glucose and plasma insulin. For insulin in particular, the impact of high local concentrations produced by the beta cells under physiological conditions will directly inhibit alpha cells and the glucagon secretion via paracrine effects within the pancreatic islets. Notwithstanding the lack of such assessment, it can be assumed that paracrine effects are similar to those of systemic insulin entering the pancreas and its islets from the arterial circulation, as in our clamp settings. Further, other metabolites, such as free fatty acids and amino acids and their derivatives can directly or indirectly influence the secretion of the investigated counter-regulatory hormones and they may thus also modify the effects of glucose and insulin. The experiments were conducted in the fasting state with standardized insulin and/glucose concentrations, but nonetheless, the interplay with such metabolites may differ between individuals.

## Conclusion

In conclusion, a novel population model was developed and applied to pooled cohorts of participants in glucose clamps. The results indicated that insulin resistance was independently associated with: i) a general increase in glucagon secretion across a broad glycemic range and ii) an increased ACTH and, consequently, cortisol response during hypoglycemia manifested by an exaggerated response to mild glucose lowering. The elevated glucagon secretion may largely be explained by alpha cell dysfunction where paracrine and neuroendocrine factors may contribute. Conversely, the ACTH/cortisol dysregulation is expected to be of central origin involving the hypothalamus and adjacent brain regions. Thus, a chronic increase in glucagon together with intermittent cortisol responses during near-hypoglycemic episodes are likely to promote and maintain insulin resistance. Therefore, the observed hormonal dysregulation may in turn also contribute to the gradual elevation of glycemia and in due course possibly to the establishment of type 2 diabetes.

## Declaration of interest

The authors declare no conflicts of interest in relation to the present work. RAE is a full-time employee of Olink Proteomics AB.

## Funding

The Swedish Diabetes Foundation, the Novo Nordisk Foundation, the Family Ernfors Foundation, EXODIAB, ALF grants of Uppsala University Hospital.

## Data availability

Datasets generated during and/or analyzed during the current study are not publicly available but are available from the corresponding author on reasonable request

## Author contribution statement

JWE designed the studies, contributed to analyses and wrote the manuscript. RAE, MHL and NA contributed to study design, data handling and analyses and reviewed/edited the manuscript. MCK researched data, contributed to study design and wrote the manuscript.
